# Postoperative radiotherapy for pediatric brain tumor: a lesson learned from treatment of a 5-year-old girl for posterior fossa astrocytoma (WHO1) in 1967

**DOI:** 10.1007/s00701-018-3636-3

**Published:** 2018-07-26

**Authors:** Tryggve Lundar, Bernt Johan Due-Tønnessen, Radek Fric, Paulina Due-Tønnessen

**Affiliations:** 1 Department of Neurosurgery, Oslo University Hospital, Oslo, Norway; 2 Faculty of Medicine, Institute of Clinical Medicine, University of Oslo, Oslo, Norway; 3 Department of Radiology, Oslo University Hospital, Oslo, Norway

Dear Editor,

We have for many years followed a patient whose long-term history illustrates how therapeutic decisions for benign lesions can affect and determine the entire life of a patient.

In 1967, a 5-year-old girl presented with progressive headache, vomiting, and ataxia. Clinical examination disclosed papilledema. Ventriculography demonstrated supratentorial hydrocephalus and lack of contrast passage to the fourth ventricle indicating a posterior fossa tumor. She underwent resection of a large, partially cystic tumor. Postoperatively, there was a right-sided hypacusis and slight persistent ataxia.

The histological examination showed astrocytoma WHO1. The resection was considered incomplete and she was given postoperative radiotherapy to the posterior fossa. Two posterior fossa fields were given small doses over 15 days up to 2750 r in each field.

She recovered and after a couple of years, someone asked: “why did you give XRT?”

The answer was: “It was not much—only 2750 r x 2”*.*

Fourteen years later, in 1981, she once more presented with clinical symptoms and signs of intracranial hypertension. A CT scan revealed a large cystic expansion in the posterior fossa along with supratentorial hydrocephalus. After a second operation with opening of the cyst and subtotal tumor resection, the histology was once more astrocytoma grade WHO1. After this second procedure, she once more recovered, but experienced right-sided anacusis and over time progressive left-sided hypacusis as well.

During the late 1990s, she deteriorated and MRI now disclosed large supratentorial meningioma formations. The tumor was resected along with the right side of the tentorium in 2000. Two years later, in 2002, she needed resection of a large meningioma in the sellar region and she was given hormonal substitution. During the following years, she lost her vision over time and underwent repeat tumor resections for aggressive malignant meningiomas in the years 2004 and 2006.

**Fig. 1:**
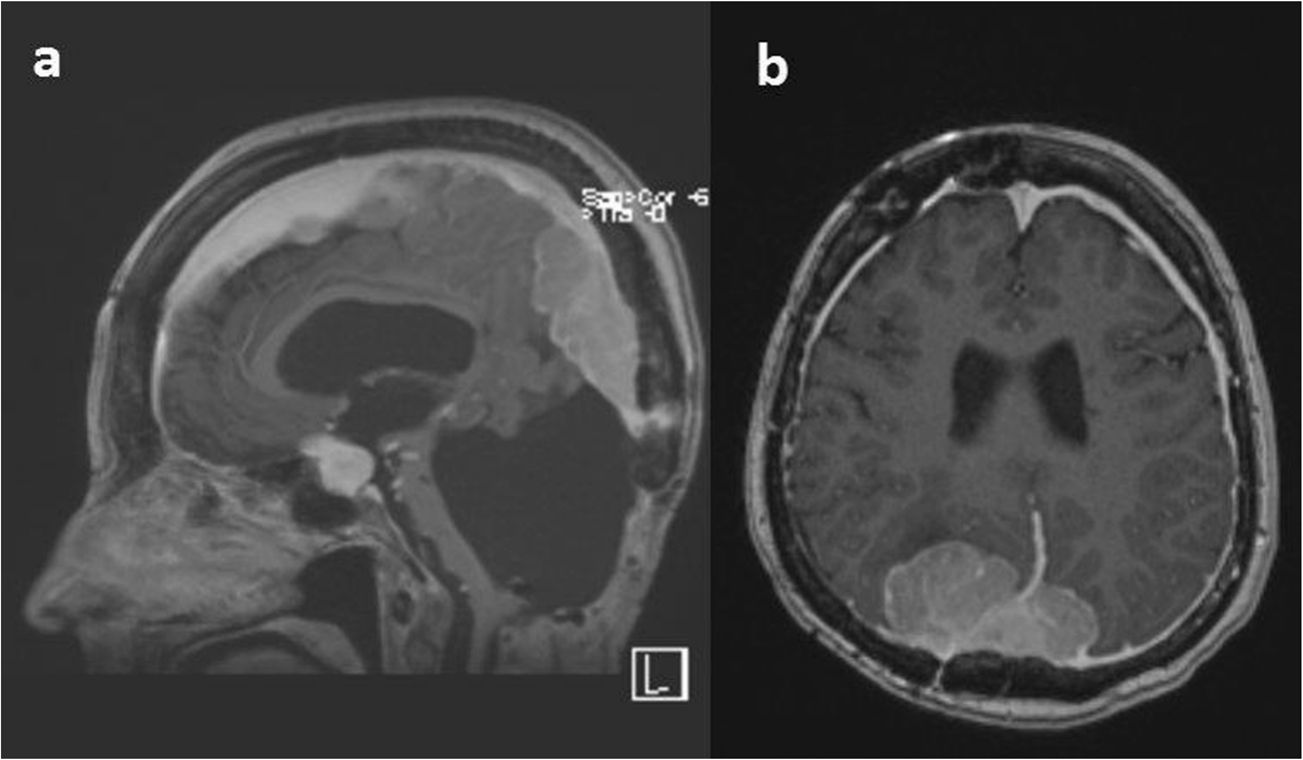
Sagittal MRI showing meningioma along the falx cerebri and the sella region, but no residual tumor in the posterior fossa (2011)

The clinical condition deteriorated further and she was bedridden, deaf, and blind during the rest of life, until she passed away in 2016. Follow-up MRI in 2011 demonstrated further intracranial meningioma progression (Fig. 1), but additional operative treatment was not found useful and she was given only palliative care until she died in 2016.

Fifty years ago, postoperative radiotherapy in the treatment of posterior fossa medulloblastoma dramatically improved the 5-year survival rates from zero to more than 50% [3]. In children with low-grade posterior fossa astrocytoma, such treatment has never been standard but has been given in selected cases. The decision for radiotherapy appears to be incomplete resection or such treatment is applied after a second resection or instead of repeat surgery [2].

Although our policy has been repeat surgery when indicated instead of adjuvant radiotherapy, a few patients have been given such adjuvant postoperative radiotherapy up to the late 1980s [1]. It is difficult to delineate the precise reason for the decision. It may reflect differences in opinion among the individual members in the team when treatment after incomplete resection was discussed. This case reminds us that a simple therapeutic decision which was apparently taken lightly had serious consequences for this patient.

The present case is—in our opinion—*a kind of the worst possible result—a progressive disaster over nearly five decades.*
